# Co-localization of zinc transporter 3 (ZnT3) with sensory neuromediators and/or neuromodulators in the enteric nervous system of the porcine esophagus

**DOI:** 10.1007/s10534-017-0014-1

**Published:** 2017-04-17

**Authors:** Joanna Wojtkiewicz, Liliana Rytel, Krystyna Makowska, Sławomir Gonkowski

**Affiliations:** 1 Department of Pathophysiology, Faculty of Medical Sciences, University of Warmia and Mazury, Olsztyn, Poland; 2 Laboratory for Regenerative Medicine, Faculty of Medical Sciences, University of Warmia and Mazury, Olsztyn, Poland; 3 Foundation for the Nerve Cells Regeneration, Warszawska Str. 30, Mazury, 10-082 Olsztyn, Poland; 4 Department of Internal Medicine and Clinic, Faculty of Veterinary Medicine, University of Warmia and Mazury, Oczapowskiego Str 15, 10-718 Olsztyn, Poland; 5 Department of Clinical Physiology, Faculty of Veterinary Medicine, University of Warmia and Mazury, Oczapowskiego Str. 13, 10-718 Olsztyn, Poland

**Keywords:** Enteric nervous system, Zinc transporters, Esophagus, Immunofluorescence technique, Pig

## Abstract

Zinc transporter 3 (ZnT3) is one of the zinc transporters family. It is closely connected to the nervous system, where enables the transport of zinc ions from the cytoplasm to synaptic vesicles. This substance has been described within the central and peripheral nervous system, especially in the enteric nervous system (ENS). The aim of the present study was to describe the co-localization of ZnT3 with selected neuromediators and/or neuromodulators participating in sensory stimuli conduction in neurons of the ENS within the porcine esophagus. Co-localization of ZnT3 with substance P (SP), leucine enkephalin (LENK) and calcitonin gene-related peptide (CGRP) was studied using standard double-immunofluorescence technique. The obtained results show that ZnT3, SP and/or LENK may occur in the same enteric neurons, and the degree of co-localization of these substances clearly depends on the fragment of esophagus studied and the type of enteric ganglia. In contrast, the co-localization of ZnT3 with CGRP was not observed during the present investigation. The obtained results suggest that ZnT3 in the ENS may be involved in the conduction of sensory and/or pain stimuli.

## Introduction

It is widely known that zinc (Zn) is one of the most important microelements in the living organism. This metal, as a key component of many proteins and co-factor of over 300 enzymes, takes part in a wide range of processes connected with cellular metabolism, including, among others, DNA synthesis, cells division and differentiation, communication between cells and immunological processes (Vallee and Auld 1990; Vallee and Falchuk 1993; Takeda 2000). Zinc ions cannot cross the biological membranes by passive diffusion, and zinc homeostasis, which is essential for correct functioning of the organism, is maintained by metallothioneins (MTs) and transmembrane transporters (Hojyo and Fukada 2016; Hara et al. 2017).

Zinc transporters are divided into two groups: the solute-linked carrier 30 (SLC 30) protein family of zinc transporters (marked in the mammals from ZnT1 to ZnT10) and the Zip (Zrt- and Irt-like proteins) family (solute-linked carrier 39-SLC39) (Hara et al. 2017). First of them take place in the transport of zinc ions from cytoplasm into the lumen of organelles or extracellular space. In turn, Zip proteins have the opposite activity and transport zinc ions from extracellular space to the organelles (Eide 2006).

From all zinc transporters, only ZnT3 seems to be so closely linked to the nervous system, where it takes part in the transport of zinc ions from the cytoplasm to synaptic vesicles. Till now ZnT3 has been mainly described within the central nervous system (Frederickson et al. 2000, 2005), where it may be involved in sensory stimuli conduction, secretory activity and/or inhibitory processes (Frederickson et al. 2005). Moreover, it is known that ZnT3 plays some functions in adaptive phenomena under pathological factors, including epilepsy, mechanical injury or ischemia (Chi et al. 2008).

Contrary to the central nervous system, the knowledge concerning distribution and functions of ZnT3 in the peripheral nervous system is very scanty (Wang et al. 2003). In the light of the previous studies, it seems that the enteric nervous system (ENS) is the part of peripheral nervous system, where ZnT3 is present in relatively high amount (Gonkowski et al. 2009; Wojtkiewicz et al. 2012a, b, c, 2016).

The ENS, located in the wall of the gastrointestinal (GI) tract, due to the high degree of independence from the brain, a large number of neurons and complex structure, is often called “the second brain”. The anatomy of the ENS depends on the fragment of the digestive tract and animal species, and in the esophagus it consists of two types of intramural ganglia: myenteric ganglia (MG) placed between the longitudinal and circular muscle layers and submucous ganglia (SG)—located near the lamina propria of the mucosa (Wojtkiewicz et al. 2016), which are interconnected with dense network of nerves. Enteric neurons control all functions of the GI tract both under physiological conditions and during pathological factors, including, among others, intestinal motility, excretive activity, mesenteric blood flow and immunological processes (Vasina et al. 2006). Cells of the ENS exhibit significant differentiation in terms of morphology, functions and electrophysiological properties, but the main criterion of enteric neurons classification is the “neurochemical coding”, in the other words, the capability to expression of specific active substances (Furness 2012; Furness et al. 2014).

Several dozen factors, which most frequently may play functions of neuromediators and/or neuromodulators, have been described in the ENS. One of them is Znt3, that has been studied in the small and large intestine of human and pigs (Gonkowski et al. 2009; Wojtkiewicz et al. 2012a, b, c). It has been also studies in the esophagus (Wojtkiewicz et al. 2016), but till now the participation of ZnT3 in sensory stimuli conduction within this part of the GI tract has not been investigated at all. So, the aim of the present study was to describe the co-localization of ZnT3 with substance P (SP), leucine enkephalin (LENK) and calcitonin gene-related peptide (CGRP), which are known as important factors involved in sensory stimuli conduction.

## Materials and methods

The present study was made on six immature sows of the Large White Polish breed at the age of 8 weeks and about 18–20 kg body weight. Pigs were kept in typical laboratory conditions adapted for this animal species. The experiment was performed incompliance with the instructions of the Local Ethical Committee for the Experiments on Animals in Olsztyn (Poland) decision number.

After 3 days of adaptive period pigs were premedicated with Stressnil (Janssen, Belgium, 75 μl/kg of body weight, i.m.), after about 30 min. euthanized using an overdose of sodium thiopental (Thiopental, Sandoz, Kundl-Rakúsko, Austria, i.v.) and perfused transcardially with 4% buffered paraformaldehyde. The fragments of cervical, thoracic and abdominal esophagus were collected from all sows. Tissues were post-fixed in the same paraformaldehyde solution, rinsed in phosphate buffer for three days and kept in 18% sucrose at 4 °C. After at least two weeks the fragments of esophagus were frozen at −23 °C and cut into 10 μm-thick sections using microtome (Microm, HM 525, Walldorf, Germany). The sections were subjected to routine double-labeling immunofluorescence technique according to method described previously by Gonkowski et al. (2013). This method in short consisted on the following stages (all actions were performed at room temperature): drying for 45 min; incubation with a blocking solution, which included 10% normal goat serum, 0.1% bovine serum albumin, 0.01% NaN_3_, Triton ×-100 and thimerozal in PBS for 1 h; overnight incubation with a mixture of two “primary” antibodies raised in different species and directed towards zinc transporter 3 and one of the aftermentioned substances e.i. substance P, CGRP or LENK; incubation (for 1 h) with species-specific antisera conjugated to FITC or biotin, which was visualized by a streptavidin-CY3 complex (the specification of primary and secondary antibodies used in the present study is shown in Table 1). The rinsing with PBS (3 × 10 min, pH 7.4) was performed between the particular stages.

**Table 1:** Specification of immune reagents used in the study: *ZnT3* zinc transporter 3, *SP* substance P, *LENK* leucine enkephalin, *CGRP* calcitonin-gene related peptide, *FITC* fluorescein isothiocyanate, *CY3* indocarbocyanine, *H* heavy chain, *L* light chain

Antisera	Code	Host species	Dilution	Supplier
Primary antibody				
ZnT3	–	Rabbit	1:600	Gift from prof. Palmiter, University of Washington, Seattle, WA, USA
SP	8450-0505	Rat	1:300	Biogenesis Inc., Poole, UK; http://www.biogenesis.co.uk
LENK	4140-0355	Mouse	1:1000	Biogenesis Inc
CGRP	T-5027	Guinea pig	1:1000	Peninsula Labs., San Carlos, CA, USA; see Bachem AG; http://www.bachem.com

Reagent			Dilution	Supplier
Secondary antibodies				
FITC-conjugated donkey-anti-mouse IgG (H + L)			1:800	Jackson, 715-095-151, West Grove, PA, USA
FITC-conjugated donkey-anti-rat IgG (H + L)			1:800	Jackson, 712-095-153
FITC-conjugated donkey-anti-guinea pig IgG (H + L)			1:1000	Jackson, 706-095-148
Biotinylated goat anti-rabbit IgG			1:1000	DAKO, E 0432, Carpinteria, CA, USA
CY3- conjugated Streptavidin			1:9000	Jackson, 016-160-084

During the present investigation the standard controls of specificity of “primary” antibodies were performed. These included pre-absorption of the particular antisera with appropriate antigens, as well as “omission” and “replacement” tests and completely eliminated immunofluorescence signals.

Two methods of the evaluation of co-localization of ZnT3 with other substances studied were used. First of them consisted of the determination what percentage of all ZnT3-LI enteric neurons were cells immunoreactive to other substances studied. To this end at least 500 ZnT3-positive cell bodies in particular types of enteric ganglia were examined for immunoreactivity to the particular neuronal factors investigated, and ZnT3-positive neurons were considered as representing 100% for all combinations. The second method was to determine what percentage of particular neuronal population (immunoreactive to SP, LENK or CGRP) were ZnT3—positive neurons. In this case at least neurons immunoreactive to SP, LENK or CGRP were evaluated for ZnT3—like immunoreactivity, and the numbers of cells immunoreactive to particular substances studied (SP, LENK or CGRP) were considered as 100%.

Double-labeled perikarya (only neurons with clearly-visible nucleus were included) were determined under an Olympus BX51 microscope equipped with epi-fluorescence and appropriate filter sets. The obtained results were pooled and presented as mean ± SEM. To prevent double counting of the same neuronal cells, the sections evaluated during the present study were located at least 100 µm apart. Statistical analysis was carried out with Student’s *t* test (Graphpad Prism v. 6.0; GraphPad Software Inc., San Diego, CA, USA). The differences were considered statistically significant at p ≤ 0.05.

## Results

During this study the presence of ZnT3—positive neurons were observed in both types of enteric ganglia of all parts of esophagus studied, and this zinc transporter co-localized in the same cells with two from among three substances studied.

**Fig. 1:**
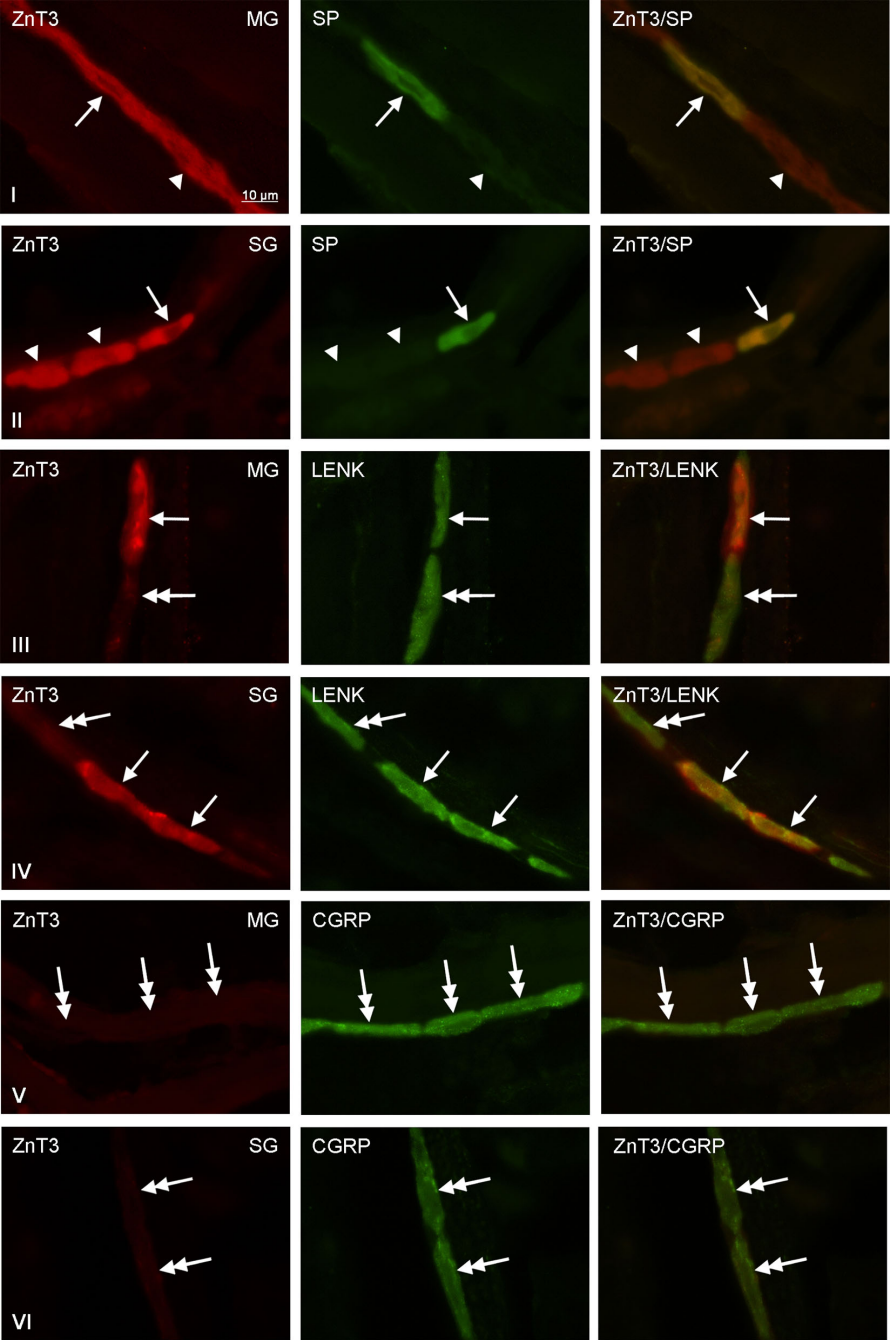
Co-localization of zinc transporter 3 (ZnT3) with sensory neuromediators and/or neuromodulators in the enteric nervous system within cervical part of the porcine esophagus: *MG* myenteric ganglia, *SG* submucous ganglia, *SP* substance P, *LENK* leucine enkephalin, *CGRP* calcitonin gene-related peptide. Neurons, where ZnT3 co-localizes with SP, LENK or CGRP are indicated with *arrows*. Neurons immunoreactive to ZnT3, but negative to SP, LENK or CGRP are indicated *arrowheads*. SP-, LENK- or CGRP-like immunoreactive neurons, where ZnT3 is not present are indicated with double-headed *arrows*. The *right* column of the pictures shows the overlap of both stainings

**Fig. 2:**
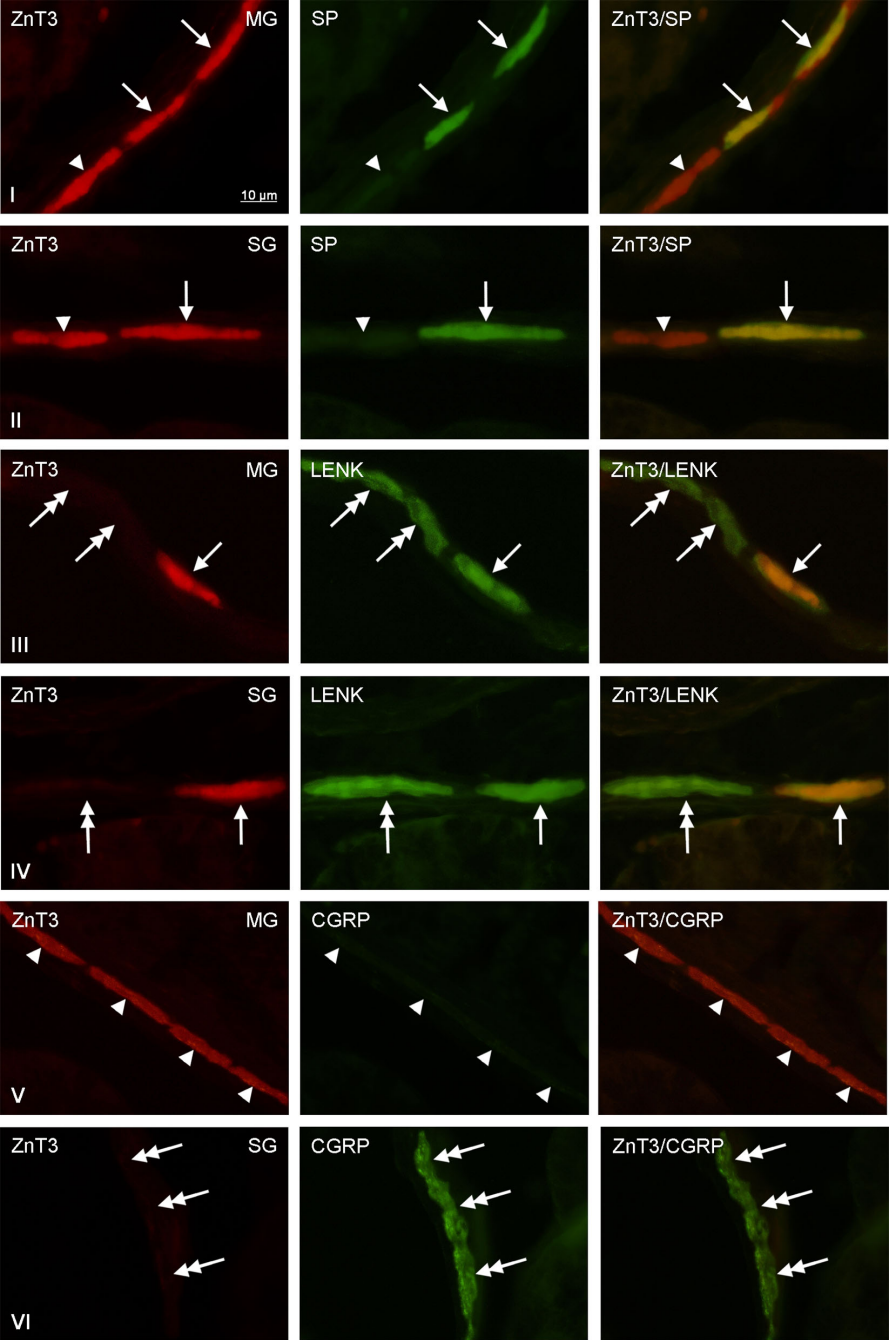
Co-localization of zinc transporter 3 (ZnT3) with sensory neuromediators and/or neuromodulators in the enteric nervous system within thoracic part of the porcine esophagus: *MG* myenteric ganglia, *SG* submucous ganglia, *SP* substance P, *LENK* leucine enkephalin, *CGRP* calcitonin gene-related peptide. Neurons, where ZnT3 co-localizes with SP, LENK or CGRP are indicated with *arrows*. Neurons immunoreactive to ZnT3, but negative to SP, LENK or CGRP are indicated *arrowheads*. SP-, LENK- or CGRP-like immunoreactive neurons, where ZnT3 is not present are indicated with double-headed *arrows*. The *right* column of the pictures shows the overlap of both stainings

**Fig. 3:**
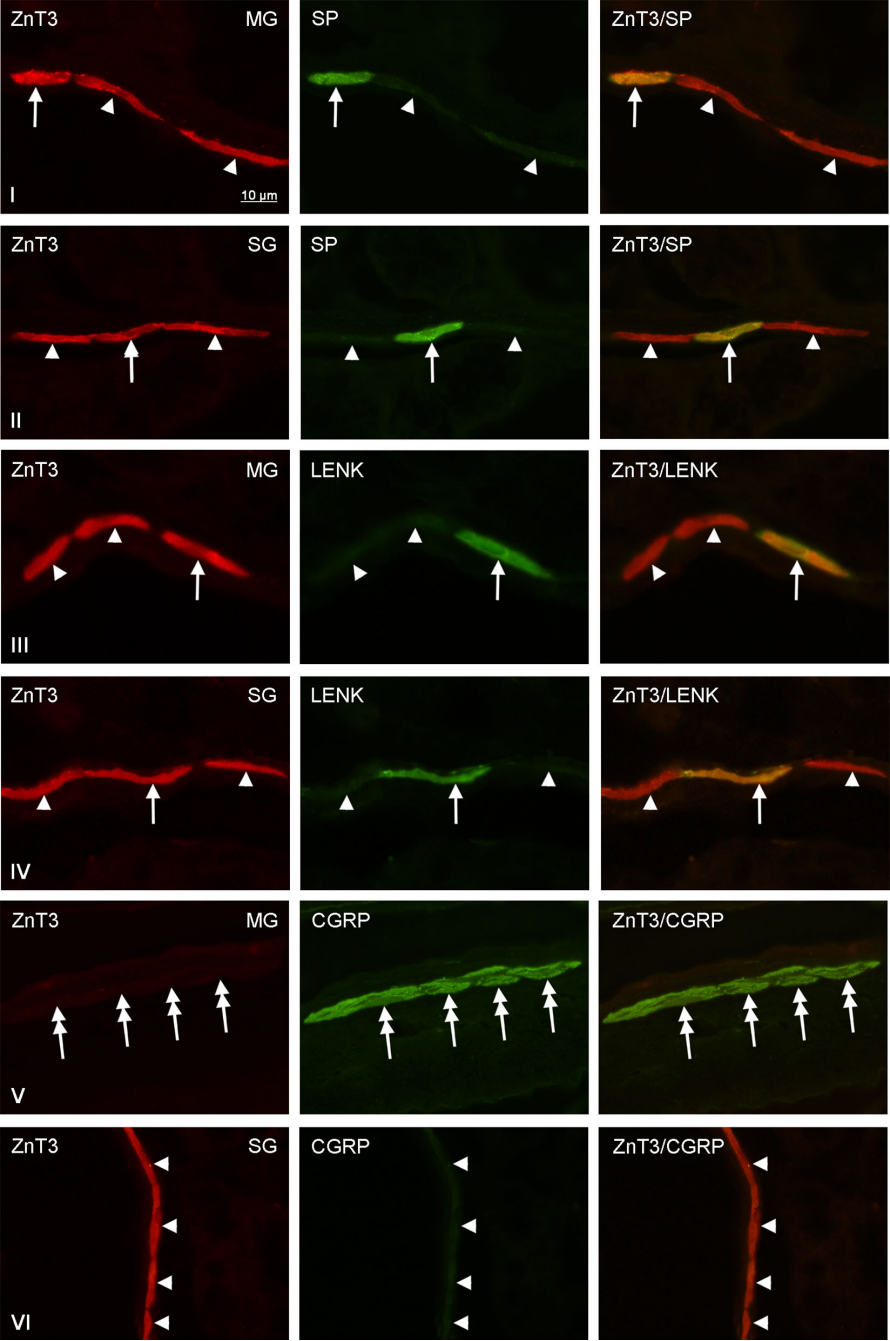
Co-localization of zinc transporter 3 (ZnT3) with sensory neuromediators and/or neuromodulators in the enteric nervous system within abdominal part of the porcine esophagus: *MG* myenteric ganglia, *SG* submucous ganglia, *SP* substance P, *LENK* leucine enkephalin, *CGRP* calcitonin gene-related peptide. Neurons, where ZnT3 co-localizes with SP, LENK or CGRP are indicated with *arrows*. Neurons immunoreactive to ZnT3, but negative to SP, LENK or CGRP are indicated *arrowheads*. SP-, LENK- or CGRP-like immunoreactive neurons, where ZnT3 is not present are indicated with double-headed *arrows*. The *right* column of the pictures shows the overlap of both stainings

Co-localization of ZnT3 with SP and/or LENK were noted in the myenteric and submucous ganglia in cervical (Fig. 1), thoracic (Fig. 2) and abdominal (Fig. 3) esophagus, and the degree of its clearly depended on both the type of enteric ganglia and esophageal fragment (Tables 2, 3). Contrary to SP and LENK, during the present investigation CGRP was not observed at all in esophageal enteric neurons immunoreactive to ZnT3 (Tables 2, 3), although on the other hand CGRP—positive enteric neuronal cells (ZnT3—negative) were observed in the esophagus (Fig. 1V, VI; 2 V, VI; 3 V, VI).

**Table 2:** The percentage (mean ± SEM) of neurons immunoreactive to leucine enkephalin (LENK), substance P (SP), and/or calcitonin gene-related peptide (CGRP) in relation to the population of ZnT3-positive neurons in the enteric nervous system of the porcine esophagus

	Myenteric ganglia	Submucous ganglia
Cervical esophagus		
ZnT3+/LENK+	80.3 ± 3.2	21.5 ± 7.7
ZnT3+/SP+	4.4 ± 1.8	1.1 ± 0.5
ZnT3+/CGRP+	0	0
Thoracic esophagus		
ZnT3+/LENK+	10.3 ± 7.0	20.0 ± 1.3
ZnT3+/SP+	21.1 ± 2.0	25.0 ± 1,5
ZnT3+/CGRP+	0	0
Abdominal esophagus		
ZnT3+/LENK+	93.5 ± 3.8	91.7 ± 3.0
ZnT3+/SP+	15.0 ± 2.0	13.8 ± 8.2
ZnT3+/CGRP+	0	0

ZnT3 positive neurons were considered as representing 100%

**Table 3:** The percentage (mean ± SEM) of zinc-transporter-3-like immunoreactive perikarya in neuronal populations immunoreactive to particular active substances studied

	Myenteric ganglia	Submucous ganglia
Cervical esophagus		
LENK+/ZnT3+	88.7 ± 3.0	72.0 ± 5.8
SP+/ZnT3+	44.3 ± 5.6	55.0 ± 4.0
CGRP+/ZnT3+	0	0
Thoracic esophagus		
LENK+/ZnT3+	75.0 ± 3.0	50.0 ± 4.0
SP+/ZnT3+	100	25.0 ± 4.0
CGRP+/ZnT3+	0	0
Abdominal esophagus		
LENK+/ZnT3+	16.6 ± 4.7	22.0 ± 6.4
SP+/ZnT3+	88.4 ± 3.2	63.3 ± 8.2
ZnT3+/CGRP+	0	0

The number of neurons immunoreactive to each substance were considered as representing 100%

In the cervical esophagus (Fig. 1) the significant differences in the degree of co-localization of ZnT3 with LENK were observed between myenteric and submucous ganglia. In the MG LENK was present within 80.3 ± 3.2% of all ZnT3—positive cells, whereas in the SG the percentage of ZnT3+/LENK+ cells in relation to all neurons immunoreactive to ZnT3 amounted to only 21.5 ± 7.7% (Table 2). Considerable lower percentage of ZnT3-positive neurons in the wall of cervical esophagus showed simultaneously the presence of SP. These values amounted to 4.4 ± 1.8 and 1.1 ± 0.5% of all ZnT3+ neurons in the MG and SG, respectively (Table 2).

In the myenteric ganglia of thoracic esophagus (Fig. 2) the degree of co-localization of ZnT3 with LENK was clearly lower than in cervical part of this organ and achieved only 10.3 ± 7.0% of all cells immunoreactive to ZnT3. In turn, within SG the presence of LENK was noted in 20.0 ± 1.3% of ZnT3+ cells and this value was similar to those observed in the SG of the cervical esophagus (Table 2). The percentage of ZnT3+/SP+ neurons in relation to all Znt3-LI cells in thoracic esophagus was levelised in both types of enteric ganglia and significantly higher than in the cervical esophagus. These values amounted to 21.1 ± 2.0% and 25.0 ± 1, 5 in the MG and SG, respectively (Table 2).

In the MG of abdominal esophagus (Fig. 3) the percentages of ZnT3+/LENK+ and ZnT3+/SP+ neurons in relation to all cells immunoreactive to ZnT3 were levelised and achieved 16.6 ± 4.7 and 15.0 ± 2.0%, respectively. In the SG of abdominal esophagus the number of ZnT3—positive cells, which simultaneously immunopositive to LENK amounted to 22.0 ± 6.4% and was similar to values observed in the cervical and thoracic esophagus. In turn the percentage of ZnT3+/SP+ neurons in relation to all Znt3—LI cells achieved 13.8 ± 8.2% and was about 50% lower than in the thoracic esophagus, but significantly higher than within the cervical part of this organ (Table 2).

The degree of percentage of ZnT3—positive cells in relation to all population of neurons immunoreactive to LENK or SP also depended on the fragment of esophagus and type of enteric ganglion (Table 3). In the cervical esophagus (Fig. 1) both in the MG and SG relatively large number of LENK-LI cells showed also the presence of ZnT3. These values amounted to 88.7 ± 3.0 and 72.0 ± 5.8% in the MG and SG, respectively. The percentage of ZnT3+ neurons in relation to the population of cells immunoreactive to LENK in both types of enteric ganglia at first clearly decreased in caudal direction, reaching in the thoracic esophagus 75.0 ± 3.0% in the MG and 50.0 ± 4.0% in the SG before increasing again to 93.5 ± 4.7% (in the MG) and 91.7 ± 3.0% (in the SG) within the abdominal part of this organ (Table 3).

In the cervical esophagus the percentage of ZnT3+ cells in relation to all SP—positive neurons amounted to 44.3 ± 5.6% in the MG and 55.0 ± 4.0% in the SG. In the MG of other part of esophagus studied these values clearly increased, especially in the thoracic esophagus, where all SP—positive cells observed during present investigations were also immunoreactive to ZnT3 (Table 3). In the MG of the abdominal esophagus the number of SP+/ZnT3+ cells in relation to all SP—positive neurons (88.4 ± 3.2%) was lower than in the thoracic fragment, but twice higher than within the cervical esophagus (Table 3). I turn, the percentage of ZnT3-LI cells in relation to all SP+ neurons studied in the SG of thoracic esophagus amounted to 25.0 ± 4.0%, and within abdominal esophagus achieved 63.3 ± 8.2%.

## Discussion

The results obtained during this study show that ZnT3 is present in the enteric neurons of all fragments of esophagus studied, what is in agreement with previous investigations (Wojtkiewicz et al. 2016). It should be pointed out that exact functions of ZnT3 within enteric neurons still remain unclear and are often described by analogy with the central nervous system, where the activity of this zinc transporter is better known. In the brain Znt3 takes part in the regulation of zinc ions levels in neuronal cells (Palmiter et al. 1996; Palmiter and Huang 2004) and it seems to be a typical substance for neurons that used zinc as a neuromodulator (Takeda et al. 2013). Moreover ZnT3 facilitates the transport of zinc from the cytoplasm to synaptic vesicles (Palmiter et al. 1996), and thereby vicariously influences on the synaptic zinc ions levels. So ZnT3 seems to be a key factor, which is involved in the maintenance of synaptic Zn^2+^ homeostasis. On the other hand, it is relatively well established that this homeostasis is essential for correct functioning of the nervous system, because both excess and deficiency of synaptic zinc ions may cause disturbances in neuronal physiology and neurodegeneration (Weiss et al. 2000; Takeda et al. 2013). Probably, the involvement of ZnT3 in the regulation of synaptic Zn2^+^ levels is at the very heart of previously described changes in the expression of this zinc transporter during various pathological processes in the brain, including ischemia Huntington’s, Alzheimer’s and Parkinson’s diseases, epilepsy and mechanical damage (Devirgiliis et al. 2007; Chi et al. 2008; Whitfield et al. 2015). Moreover, it is known that ZnT3 in the central nervous system may take part in regulatory processes connected both with sensory stimuli conduction and secretory activity of neuronal cells (Danscher et al. 2001, 2003), and some previous studies view ZnT3 as the marker of inhibitory nerve fibers called zinc enriched nerves (ZEN) (Jo et al. 2000).

One could only conjecture that Znt3 in the enteric nervous system may play similar functions. Previous studies showed that this zinc transporter is present in various types of enteric neurons and co-localizes with a wide range of other neuronal substances (Wojtkiewicz et al. 2012b, c, 2016), what can suggest miscellaneous functions of this substance in the ENS. In turn, the results obtained in the present study, where co-localization of ZnT3 with LENK and/or SP was observed, can suggest involvement of this zinc transporter in sensory stimuli conduction within the gastrointestinal tract. Two facts enforces this thesis. Firstly, both SP and LENK are factors, that often are present in sensory neurons and their role in sensory and pain conduction is commonly known (De Schepper et al. 2004; Shimizu et al. 2008). Moreover, it is relatively established that substances co-localized in the same neuronal cells usually play similar functions (Wojtkiewicz et al. 2012a). Secondly, ZnT3 has been previously described as the factor, that can take part in sensory neurons activity within the central nervous system (Danscher et al. 2001).

On the other hand, it should be pointed out that the contribution of ZnT3 in sensory conduction in the ENS are not completely clear. Neither in the esophagus (results of the present study), nor in other fragments of the gastrointestinal tract (Wojtkiewicz et al. 2012b, c) the co-localization of ZnT3 with CGRP has not been observed, and CGRP seems to be one of the main sensory neurofactors in the ENS and by some investigators is considered to be the marker of the intrinsic primary afferent neurons—enteric sensory neurons and afferent component the short, intestinal intramural reflex arcs (Mawe and Sharkey 2016).

Absence of the co-localization of ZnT3 and CGRP may suggest on the one hand that CGRP is not the marker of intrinsic primary afferent neurons in the pig, and on the other hand that the co-localization observed during the present study affects not only sensory neurons. It is all the more probably because LENK and SP, apart from commonly known involvement in the sensory conduction, may play other various roles in the ENS. Namely, SP influences the intestinal motility, and the character of this activity depends on animal species and the fragment of the digestive tract. For example SP strongly induces intestinal muscles contractions in rats and dogs (Lördal et al. 1993; Thor et al. 1982), contrary to the human, where this activity of SP is rather infinitesimal (Lördal et al. 1997). Moreover, SP in the ENS also regulates the excretive activity of the digestive tract and mesenteric blood flow (Brunsson et al. 1995), as well as takes part in adaptive and neuroprotective processes under pathological factors (Shimizu et al. 2008; Gonkowski 2013). In turn, LENK participates in inhibition of intestinal motility and secretion and takes part in immunological activity of the GI tract (Puig and Pol 1998; Pol et al. 2003; De Schepper et al. 2004).

Moreover, the degree of co-localization of ZnT3 with SP and/or LENK observed during the present study significantly differs from those observed in other parts of the porcine digestive tract (Wojtkiewicz et al. 2012b, c), what may suggest that exact roles of both ENS as a whole, as well as ZnT3 as an active substance clearly depend on the fragment of the digestive tract.

To sum up, the results obtained during the present study strongly suggest that ZnT3 in the ENS of the porcine esophagus may take part in the sensory and pain stimuli conductions. On the other hand some aspects connected with this role of ZnT3 remain unclear and, due to complicated construction of the ENS, as well as omnidirectional activities of this zinc transporter, require the further investigations.
